# Humoral and cellular immune responses to Pfizer-BioNTech BNT162b2 SARS-CoV-2 vaccine in adolescents with liver transplantation: Single center experience

**DOI:** 10.3389/fimmu.2022.1049188

**Published:** 2022-11-23

**Authors:** Elena Sánchez-Zapardiel, María Alós, Pilar Nozal, Miguel González-Muñoz, Esteban Frauca-Remacha, Lucía Blanca Gavilán, María José Quiles, Loreto Hierro, Eduardo López-Granados

**Affiliations:** ^1^ Department of Immunology, La Paz University Hospital, Madrid, Spain; ^2^ Lymphocyte Pathophysiology in Immunodeficiency Group, La Paz Biomedical Research Institute (IdiPAZ), La Paz University Hospital, Madrid, Spain; ^3^ European Reference Network (ERN) Transplant-Child, Madrid, Spain; ^4^ Department of Pediatric Hepatology, La Paz University Hospital, Madrid, Spain; ^5^ Diagnosis and Treatment of Pathologies Associated with Alterations of the Complement System Group, La Paz Biomedical Research Institute (IdiPAZ), La Paz University Hospital, Madrid, Spain; ^6^ Rare Diseases Networking Biomedical Research Centre​ (CIBERER U754), Madrid, Spain; ^7^ Patient Safety and Quality Research Group, La Paz Biomedical Research Institute (IdiPAZ), La Paz University Hospital, Madrid, Spain; ^8^ Rare Diseases Networking Biomedical Research Centre​ (CIBERER U767), Madrid, Spain

**Keywords:** BNT162b2 SARS-CoV-2 vaccine, adolescent liver transplantation, SARS-CoV-2-specific IgG antibodies, IFN-γ release assay, COVID-19

## Abstract

**Background:**

Immune responses to vaccines against severe acute respiratory syndrome (SARS)-coronavirus (CoV)-2 are variable. In the absence of disease, youngsters are expected to better react to vaccines than adults. Nevertheless, chronic immunosuppression in transplant recipients may impair their capability to generate protection. We aim to explore immune responses after BNT162b2 SARS-CoV-2 vaccination in our cohort of young liver-transplanted patients.

**Methods:**

A prospective study of adolescent liver-transplanted patients (n=33) in the long-term follow-up was performed. Immune responses after receiving Pfizer-BioNTech BNT162b2 vaccine were analyzed at two time-points: baseline and 30 days after the second dose. Humoral responses were measured by fluoroenzyme-immunoassay and T-cell responses by interferon-γ-release assay. Post-vaccine coronavirus disease (COVID-19) events were recorded by a survey.

**Results:**

Pre-vaccine SARS-CoV-2-specific antibodies were undetectable in 27/32 (84.4%), negative/indeterminate in 3/32 (9.4%) and positive in 2/32 (6.3%) patients. Cellular responses at baseline were negative in 12/18 (66.6%), positive in 3/18 (16.6%) and indeterminate in 3/18 (16.6%) recipients. None of the baseline positives recalled any symptoms. Post-vaccine antibodies were detected in all patients and 92.6% showed levels >816 BAU/mL. Twenty (71.4%) recipients had positive T-cell responses. Regarding post-vaccine SARS-Cov-2 infection, 10 (30.3%) patients reported COVID-19 without hospitalization and 21 (63.6%) did not notify any infection. Negative and positive cell-response groups after vaccination showed statistically significant differences regarding COVID-19 cases (62.5% vs 22.2%, respectively; p=0.046).

**Conclusions:**

Adolescents and young adults with liver transplantation responded to SARS-Cov-2 vaccine, generating both humoral and cellular responses. Recipients developing cellular responses after vaccination had a lower incidence of COVID-19.

## Introduction

Pfizer-BioNTech BNT162b2 vaccine against severe acute respiratory syndrome (SARS)-coronavirus (CoV)-2 was the first to receive emergency use authorization from the Food and Drug Administration on 11th December 2020, for coronavirus disease (COVID-19) prevention in individuals 16 years of age or older. On May 10th 2021, emergency use authorization was expanded to include 12 years of age or older, on the basis of the data reported by Frenck et al. (1). Afterwards, other vaccines against SARS-CoV-2 were also approved (2).

Although vaccines commonly elicit complete immune responses, immunosuppressed patients may mount a weak immune response to the initially proposed two-dose scheme of anti-SARS-CoV-2 messenger RNA vaccines, as observed in recipients of solid-organ transplants (3, 4). Among liver transplant recipients, Pfizer-BioNTech BNT162b2 SARS-CoV-2 vaccine reportedly elicited inferior immunity than in healthy controls (5). Authors observed that less than half of the transplanted patients developed sufficient antibody levels against the virus and, those who did, showed two times less than the average achieved in healthy controls. Factors predicting non-response were older age, renal function and immunosuppressive treatments. More recently, higher seroconversion percentage (63%) has been reported by Ruether et al., in a prospective cohort of 194 patients, including 141 liver transplant recipients and 53 cirrhotic patients with Child-Pugh class A to C (6).

On the other hand, it has been observed that pediatric solid-organ recipients show a higher percentage (73.3%) of antibody response to vaccines than adults (7). In a recent publication, Sintusek et al. (8) studied the safety and immune response to BNT162b2 SARS-CoV-2 vaccine in a cohort of 16 liver-transplanted and 27 healthy adolescents. These authors concluded that the administration of two doses of this vaccine was safe, but less effective against the omicron variant in liver-transplant recipients than healthy individuals, since they developed weaker cellular responses. Thus, our aim is to explore humoral and cellular responses after Pfizer-BioNTech BNT162b2 SARS-CoV-2 vaccination in our cohort of young liver-transplanted patients.

## Methods

### Patients and samples

Our cohort included 33 adolescents and young-adult liver transplanted patients (age 11-21 years, median 16) in the long-term follow-up (9.9 ± 5.7 years, median 12) from La Paz University Hospital, Madrid (Spain). Ninety-one percent (n=30) of patients received tacrolimus-based immunosuppression (4.3 ± 1.4 ng/ml baseline levels), 88% patients were using corticosteroids and 76% (n=25) showed normal graft function (**Table 1**).

**Table 1:** Epidemiologic and clinical features in our cohort of young-adult liver transplanted patients.

Characteristics	Young-adult liver transplanted patients (n=33)
**Male/Female, n**	20/13
**Original disease, n** Biliary atresia Re-transplanted biliary atresia Alagille syndrome Metabolic disorders (MSUD, PA, OTCD) LKM-positive autoimmune hepatitis Hepatoblastoma Alpha-1 antitrypsin deficiency Congenital hepatic fibrosis Acute liver failure Cirrhosis	14 5 3 3 1 1 2 1 2 1
**Post-transplant time, years**	10.2 ± 5.6 (0.12-18.7)
**Tacrolimus based immunosuppression, n**	30
**Tacrolimus blood levels, ng/ml**	4.3 ± 1.4
**Treatment combinations, n** Tacrolimus Pred + Tacrolimus Pred + Tacrolimus + MMF Pred Pred + Cyclosporine	4 20 6 1 2
**Dysfunctional/Normal graft, n**	8/25
**Median ALT, U/L**	28

ALT, Alanine aminotransferase; MMF, mycophenolate mofetil; MSUD, Maple syrup urine disease; OTCD, Ornithine transcarbamylase deficiency; PA, Propionic academia; Pred, low dose methylprednisolone.

Patients received two doses of 30 µg BNT162b2, Comirnaty^®^ (Pfizer/BioNTech), 21 days apart. First doses were administered on May 2021 in recipients older than 15 years and on July 2021 in patients older than 11 years. On the second week of February 2022 (first omicron-variant wave remission), a clinical survey was completed by each patient to collect SARS-CoV-2 infection events after vaccination.

**Figure 1:**
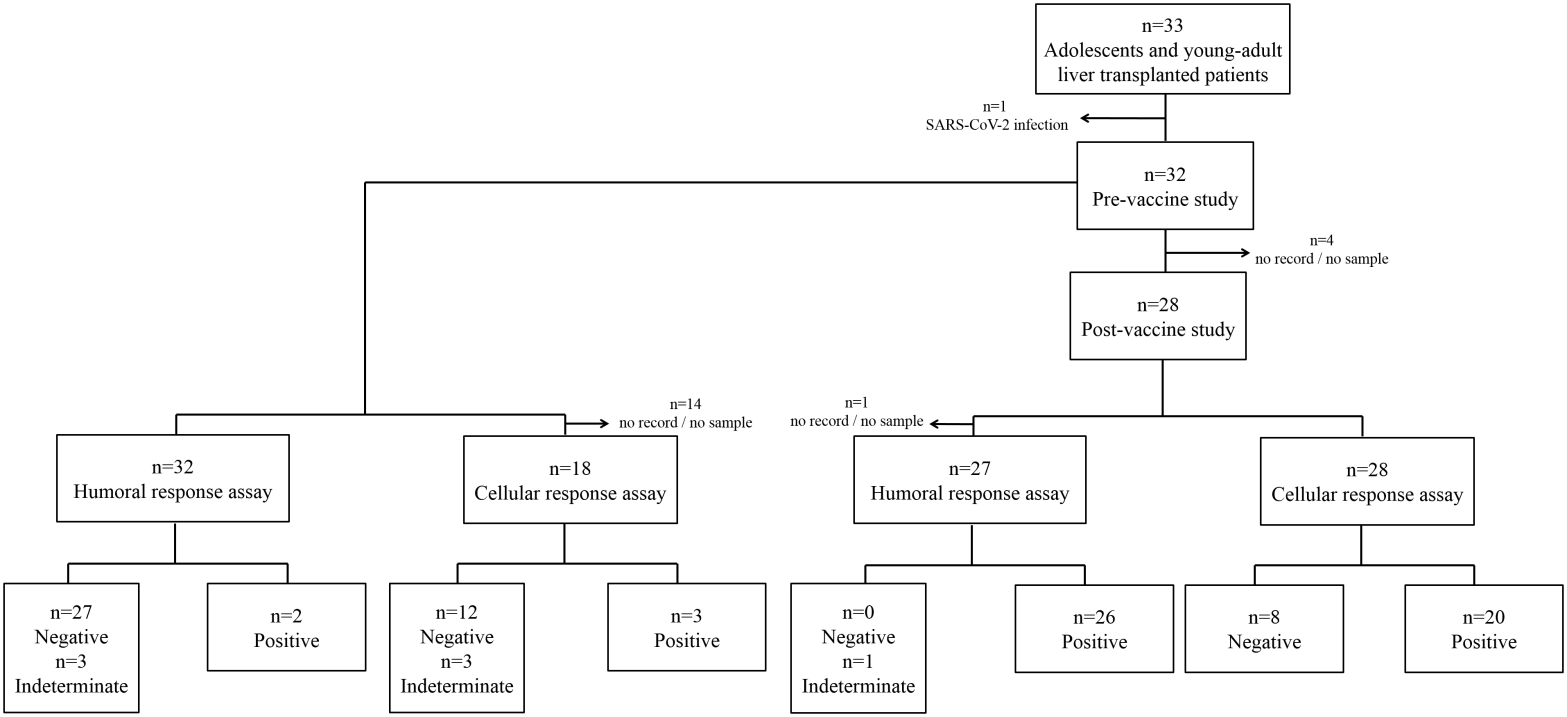
Flow diagram for the analysis of adolescent and young liver-transplanted patient cohort (n=33). First and second levels show pre- and post-vaccination groups, respectively. Levels 3-4 show the groups of humoral and cellular assays.

We analyzed humoral and cellular immune responses at two different time-points: baseline and 30 days after the second dose (**Figure 1**). We retrospectively analyzed absolute lymphocyte counts from the hemogram closer to these two timepoints: median 11 [interquartile range (IQR) 0-57] days before the first dose and 45 [IQR 27-72] days after the second dose, respectively.

### SARS-CoV-2-specific T-cell response

Briefly, 1mL of blood was drawn into manufacturer-specific tubes (QuantiFERON^®^ Human interferon gamma (IFN-γ) SARS-CoV-2, Qiagen^®^), incubated at 37°C for 16-24h and centrifuged to separate plasma. IFN-γ (IU/ml) was measured in these plasma samples by ELISA test (QuantiFERON^®^ Human IFN- γ SARS-CoV-2, Qiagen^®^). Samples were considered positive for T-cell response when exceeding the cut-off value of 0.015 IU/mL (9) in one or both antigen tubes: tube 1, containing epitopes derived from the S1 subunit of the S protein, activating T CD4+ cells; and tube 2, covering epitopes from the S1 and S2 subunits of the S protein, inducing both T CD4+ and CD8+ cell activation.

### SARS-CoV-2-specific IgG-antibody response

SARS-CoV-2-specific IgG antibodies against spike antigen were measured in 100μL of human serum samples by fluoroenzyme-immunoassay (EliA SARS-CoV-2-Sp1 IgG Test, Thermo Fisher Scientific) using the Phadia 250 instrument. The cut-off value was set at 40 BAU/mL (lower limit of equivocal zone set to 28 BAU/mL). The lower detection limit was 2.8 BAU/mL and the upper detection limit was 816 BAU/mL. Samples above that value were not subsequently diluted. Cut-off value, lower and upper detection limits were set by the manufacturer and replicated in a cohort of healthy donors, pre- and post-vaccination, including pre-infected individuals.

### Statistical analysis

Descriptive data are presented as mean ± standard deviation (SD) and the respective range. Non-Gaussian variables are presented as median with IQR. Categorical data are presented as absolute number and proportion (%). Significance of differences comparing frequencies was determined by Fisher’s exact test or Pearson *χ^2^*-test (with Yate’s correction for continuity) and by *t* test or analysis of variance (Kruskal-Wallis) when comparing mean values. P-values under 0.05 were considered significant. The software packages SPSS (version 18.0), GraphPad Prism (version 5.02) and MedCalc^®^ (version 13.3.0) were used for statistical analysis.

## Results

### Pre-vaccination

Before sample extraction, one patient was infected with SARS-CoV-2 and, consequently, was excluded from the study (**Figure 1**). SARS-CoV-2-specific IgG antibodies were undetectable in 27 (84.4%), negative or indeterminate in 3 (9.4%) and positive in 2 (6.3%) patients at basal timepoint (**Figure 1**). On the other hand, cell responses were negative in 12 (66.6%), positive in 3 (16.6%) and indeterminate in 3 (16.6%) recipients at baseline (**Figure 1**). Remarkably, one patient had both positive antibody and cellular responses pre-vaccination. One of 2 indeterminate-antibody patients yielded a positive cellular response. None of the positives recalled any clinical symptoms of infection.

Median absolute lymphocyte counts was not statistically different pre-vaccination and after receiving the second dose (2.35 [IQR 1.68-3.00] vs 1.89 [IQR 1.49-2.84] x10^3^/μL, respectively; p=0.16).

### Post-vaccination

Antibodies were detected in all patients (**Figure 1**), with levels higher than 816 BAU/mL in 25/27 (92.6%) recipients. One individual showed low antibody levels, 10.4 BAU/mL, not explained by any particular characteristic. Cellular response was positive in 20/28 (71.4%) cases (**Figure 1**). Patients with negative and positive cellular responses showed no differences in absolute lymphocyte counts (1.55 [IQR 1.41-3.01] vs 1.92 [IQR 1.81-2.63] x10^3^/μL, respectively; p=0.79).

Comparison between patients developing and non-developing a cellular response to vaccination did not yield any statistically significant difference. We considered variables such as gender, original disease, age, post-liver transplantation time, graft function, immunosuppression combination, tacrolimus level, days after vaccine and Epstein-Barr viral load in blood.

Regarding SARS-Cov-2 infection after vaccination, 10 (30.3%) patients reported COVID-19 without hospitalization, 21 (63.6%) individuals were not infected and 2 (6.1%) did not answer the clinical survey. Seventy percent of infections (7/10) occurred during the first omicron-variant wave. Comparing negative and positive cell-response groups after vaccination, the only variable rendering statistically significant differences was percentage of COVID-19 cases (62.5% [5/8] vs 22.2% [4/18], respectively; p=0.046), without relation with severity of symptoms. **Table 2** summarizes other potential cofounders between negative and positive cell-response groups.

**Table 2:** Epidemiologic and clinical features in patients with negative and positive cell responses after receiving the second vaccine dose.

Characteristics	Negative cell response (n=8)	Positive cell response (n=20)	p-value
**Male/Female, n**	4/4	11/9	ns
**Age, n** < 12 years > 12 years	4 4	5 5	ns
**Post-transplant time, years**	14.2 [IQR 8.4-15.4]	12.6 [IQR 7.3-15.8]	ns
**Tacrolimus blood levels, ng/ml**	4.7 [IQR 4.7-5.3]	3.9 [IQR 3.2-4.6]	ns
**Treatment combinations, n** Tacrolimus Pred + Tacrolimus Pred + Tacrolimus + MMF Other	1 6 1 0	2 12 3 5	ns
**Dysfunctional graft, n**	1	5	ns

IQR, interquartile range; MMF, mycophenolate mofetil; ns, statistically not significant; Pred, low dose methylprednisolone.

**Figure 2:**
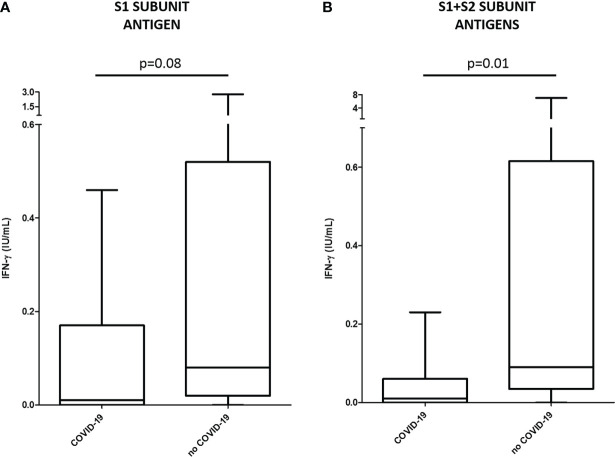
Median IFN-γ levels (IU/mL) and interquartile ranges by ELISA test (QuantiFERON^®^) in patients who reported post-vaccine SARS-CoV-2 infection (COVID-19, n=9) and individuals who were not infected (no COVID-19, n=17). Cellular response was measured by stimulating with peptides derived from the S1 **(A)** or S1 and S2 **(B)** subunits of the S protein.

Median IFN-γ levels in patients who were not infected (n=17) after the second dose were significantly higher compared to individuals who reported post-vaccine SARS-CoV-2 infection (n=9). Interestingly, differences did not reach statistical significance when T cells were stimulated with peptides derived from the S1 subunit of the S protein alone (median IFN-γ 0.08 [IQR 0.02-0.40] IU/mL in COVID-19 negative group vs 0.01 [IQR 0.00-0.05] IU/mL in COVID-19 positive group, p=0.08; **Figure 2A**), but were statistically significant when both S1 and S2 subunit-derived peptides were used (median IFN-γ 0.09 [IQR 0.05-0.55] IU/mL in COVID-19 negative group vs 0.01 [IQR 0.00-0.05] IU/mL in COVID-19 positive group, p=0.01; **Figure 2B**).

## Discussion

In our cohort, adolescents and young-adults with liver transplantation who received Pfizer-BioNTech BNT162b2 SARS-CoV-2 vaccination generated both humoral and cellular responses. Vaccine was well tolerated by all patients and graft function was unaffected. During the follow-up, liver biochemistry was normal in 87.5% cases and immune-mediated liver injury following vaccination was not suspected (10).

We reported higher antibody response to BNT162b2 vaccine in young liver-transplanted recipients compared to adult cohorts, as observed by Sintusek et al. (8). Similar to the observation by these authors, all participants in our study showed antibody response, compared to 73.3% reported by Qin et al. (7) in solid-organ transplanted children. Particular immunosuppression regimens among different solid-organ transplant programs and changes in methodology detecting antibodies could explain these differences.

Compared to other immunosuppressed patients, seroconversion rate in our cohort of adolescent and young liver-transplant recipients is 100%, in contrast to 46-85% in hematologic patients (11) or 50% in individuals with systemic inflammatory diseases who were receiving rituximab (12). As expected, the administration of B-cell depleting treatments is critical to generate a complete immune response. Hadjadj et al. (12) also observed that 95% of sera from patients treated with rituximab did not neutralize SARS-CoV-2 alpha and delta variants. Positive IgG serology against SARS-CoV-2 in our study include both neutralizing and non-neutralizing antibodies, as a functional antibody testing was not performed. Bechetti et al. (13) concluded that neutralizing ability is associated with the presence of anti-nucleocapsid antibodies, in a cohort of adult liver-transplanted patients. These authors ask for caution when interpreting the presence of antibody levels against the S protein of SARS-CoV-2, since those results can overestimate the ability of neutralizing the virus.

Interestingly, we observed that patients who developed cellular response after vaccination had a lower incidence of COVID-19 during the first omicron-variant wave and showed significantly higher levels of T cell activation in response to S-protein derived peptides. In a similar cohort of patients, Sintusek et al. (8) described lower T-cell response against the S protein in liver-transplanted adolescents than healthy controls, although the association of IFN-γ-secreting T cells with disease prevention and severity in infected participants will be part of further assessments. Nonetheless, robust T-cell activation likely contributes to a significant protection against hospitalization or death, as suggested by current observations (14). This is a major point, since people who have developed poor T-cell responses after vaccination may benefit from optimized vaccine designs. In our study, all patients received additional COVID-19 vaccine boosters (third and fourth doses), as recommended by local Health Authorities.

In our cohort, cellular responses were detectable in a lower percentage than humoral responses (71.4% vs 96.3%, respectively), as previously described (8). Differences may be explained because the techniques detecting both types of responses show distinctive sensitivity and specificity. Moreover, in this study T-cell reaction was measured through the production of a single cytokine (IFN-γ release assay or IGRA), which is preferentially secreted by cytotoxic T CD8+ cells. Polyfunctional SARS-CoV-2 T-cell profiles are commonly found in response to these vaccines (15), so detection by intracellular cytokine staining (ICS) might allow wider combination of cytokines, in addition to the capacity of stimulating cells with non-spike antigens (M and N peptides). On the other hand, IGRA tests show advantages over other cellular assays, such as ELISPOT or ICS itself, since they can be applied to a larger number of samples and easily automatized, which favors its introduction in routine clinical practice (16).

Regarding baseline immune response against SARS-CoV-2, two patients showed positive or indeterminate virus-specific IgG and positive T-cell responses pre-vaccination. It has been previously described cross-reaction of SARS-CoV-2 with other coronaviruses (17). Memory activation to specific non-spike proteins have been associated to recent infection, differentiating from pre-existing immunity in exposed populations (18). Accordingly, pre-vaccination positive reactions in our cohort might be considered as a potential consequence of previous exposure to coronaviruses, including unnoticed/subclinical SARS-CoV-2 infection. In a cohort of adult healthy donors, we observed that negative samples with detectable antibodies (2.8-28 BAU/mL) corresponded to patients who had previous contact with SARS-CoV-2 (data not shown).

The main limitations of our study are the small sample size and the lack of an age-paired immunocompetent cohort, to establish comparisons. Additionally, we acknowledge that subclinical ongoing or weeks before infections may have influenced our results.

Our findings suggest that adolescent with liver transplantation are able to mount robust immune responses to SARS-CoV-2 vaccination. As expected for younger cohorts, our patients showed higher percentages of immune responses when compared to older liver-transplanted recipients (6–8). The more information we have about the immune response against SARS-CoV-2 in immunosuppressed transplanted patients, the better we can establish the risk of developing disease following infection (19). Thus, multicenter studies would help to increase the number of cases and validate our results.

## Data availability statement

The raw data supporting the conclusions of this article will be made available by the authors, without undue reservation.

## Ethics statement

The studies involving human participants were reviewed and approved by Clinical Research Ethics Committee, University Hospital La Paz. Written informed consent to participate in this study was provided by the participants’ legal guardian/next of kin.

## Author contributions

LH and EL-G participated in the research design and writing of the article. ES-Z, MA, PN, MG-M, and EF-R participated in the data analysis and writing of the article. LBG and MJQ collected the information of patients for the databases. All authors contributed to the article and approved the submitted version.

## Funding

This research received no specific grant from any funding agency in the public, commercial, or not-for-profit sectors.

## Acknowledgments

The authors are grateful to all participating patients and their families. We would like to acknowledge the contributions of all personnel at the Department of Immunology, La Paz University Hospital, Madrid, Spain.

## Conflict of interest

The authors declare that the research was conducted in the absence of any commercial or financial relationships that could be construed as a potential conflict of interest.

## Publisher’s note

All claims expressed in this article are solely those of the authors and do not necessarily represent those of their affiliated organizations, or those of the publisher, the editors and the reviewers. Any product that may be evaluated in this article, or claim that may be made by its manufacturer, is not guaranteed or endorsed by the publisher.
